# Targeting PFKFB3 alleviates cerebral ischemia-reperfusion injury in mice

**DOI:** 10.1038/s41598-019-48196-z

**Published:** 2019-08-12

**Authors:** Olga Burmistrova, Ana Olias-Arjona, Rebeca Lapresa, Daniel Jimenez-Blasco, Tatiana Eremeeva, Dmitry Shishov, Sergei Romanov, Kristina Zakurdaeva, Angeles Almeida, Peter O. Fedichev, Juan P. Bolaños

**Affiliations:** 1 Gero Discovery LLC, Moscow, Russia; 2 Institute of Functional Biology and Genomics (IBFG), Universidad de Salamanca, CSIC, Salamanca, Spain; 3 Institute of Biomedical Research of Salamanca (IBSAL), Hospital Universitario de Salamanca, Universidad de Salamanca, Salamanca, Spain; 4 Centro de Investigación Biomédica en Red de Fragilidad y Envejecimiento Saludable (CIBERFES), Madrid, Spain; 5 Nanosyn, Inc., Santa Clara, CA 95051 USA

**Keywords:** Cellular neuroscience, Stroke

## Abstract

The glycolytic rate in neurons is low in order to allow glucose to be metabolized through the pentose-phosphate pathway (PPP), which regenerates NADPH to preserve the glutathione redox status and survival. This is controlled by 6-phosphofructo-2-kinase/fructose-2,6-bisphosphatase-3 (PFKFB3), the pro-glycolytic enzyme that forms fructose-2,6-bisphosphate, a powerful allosteric activator of 6-phosphofructo-1-kinase. In neurons, PFKFB3 protein is physiologically inactive due to its proteasomal degradation. However, upon an excitotoxic stimuli, PFKFB3 becomes stabilized to activate glycolysis, thus hampering PPP mediated protection of redox status leading to neurodegeneration. Here, we show that selective inhibition of PFKFB3 activity by the small molecule AZ67 prevents the NADPH oxidation, redox stress and apoptotic cell death caused by the activation of glycolysis triggered upon excitotoxic and oxygen-glucose deprivation/reoxygenation models in mouse primary neurons. Furthermore, *in vivo* administration of AZ67 to mice significantly alleviated the motor discoordination and brain infarct injury in the middle carotid artery occlusion ischemia/reperfusion model. These results show that pharmacological inhibition of PFKFB3 is a suitable neuroprotective therapeutic strategy in excitotoxic-related disorders such as stroke.

## Introduction

Glycolysis is widely considered a pro-survival metabolic pathway because it meets the energy needs of cells during mitochondrial bioenergetic stress^1^. However, in the brain tissue, different cell types show distinct metabolic preferences^2–4^. For instance, the metabolic use of glucose through glycolysis in neurons is normally very low, being mainly metabolized through the pentose–phosphate pathway (PPP), a metabolic route that contributes to the maintenance of neuronal redox status^5–8^. Astrocytes, in contrast, mainly obtain their cell energy needs from glycolysis, providing lactate as an oxidizable metabolic fuel to neurons^9^, which obtain energy mainly by the oxidative phosphorylation^4^.

A key factor that determines these metabolic features is 6-phosphofructo-2-kinase/fructose-2,6-bisphosphatase-3 (PFKFB3), a pro-glycolytic enzyme that is normally absent in neurons but abundant in astrocytes^7^. PFKFB3 activity produces fructose-2,6-bisphosphate (F2,6BP), a potent positive effector of the rate-limiting glycolytic enzyme, 6-phosphofructo-1-kinase (PFK1)^10,11^. The absence of PFKFB3 protein in neurons is due to its continuous degradation after ubiquitylation by the E3 ubiquitin ligase anaphase-promoting complex/cyclosome-Cdh1 (APC/C-Cdh1)^7^. In fact, APC/C-Cdh1 activity is higher in neurons than in astrocytes^7^. Notably, under certain neuropathological conditions, such as during excitotoxicity, the activity of APC/C-Cdh1 in neurons is inhibited^12^, which allows PFKFB3 protein stabilization in these cells^13^. Active neuronal PFKFB3 then stimulates glucose consumption through glycolysis, which results in a concomitant decreased PPP to cause redox stress and, eventually, apoptotic death^13^.

Stroke is the leading neurologic cause of morbidity and mortality in developed countries^14^. While the molecular mechanisms underlying this complex pathological condition are not yet completely understood, a large body of experimental data suggest that excitotoxicity, leading to mitochondrial dysfunction and increased reactive oxygen species (ROS) are contributing factors^15–17^. Therefore, it appears reasonable that ameliorating the cascade of events triggered by excitotoxic stimuli might be a promising therapeutic strategy against stroke. Accordingly, we reasoned whether pharmacological inhibition of PFKFB3, by preventing the redox stress associated with glycolytic activation, would protect neurons from the apoptotic death upon excitotoxic insults. Here, we report that small molecule inhibitor of PFKFB3 is able to protect against the apoptotic death caused by excitotoxic stimuli and an oxygen-glucose deprivation (OGD)/reoxygenation model in mouse primary cortical neurons. Furthermore, we show that *in vivo* administration of this PFKFB3 inhibitor protects against motor discoordination, neurological deficiency and brain damage in a mouse model of brain ischemia/reperfusion.

## Results

### *In vitro* characterization of two PFKFB3 inhibitors

**Figure 1:**
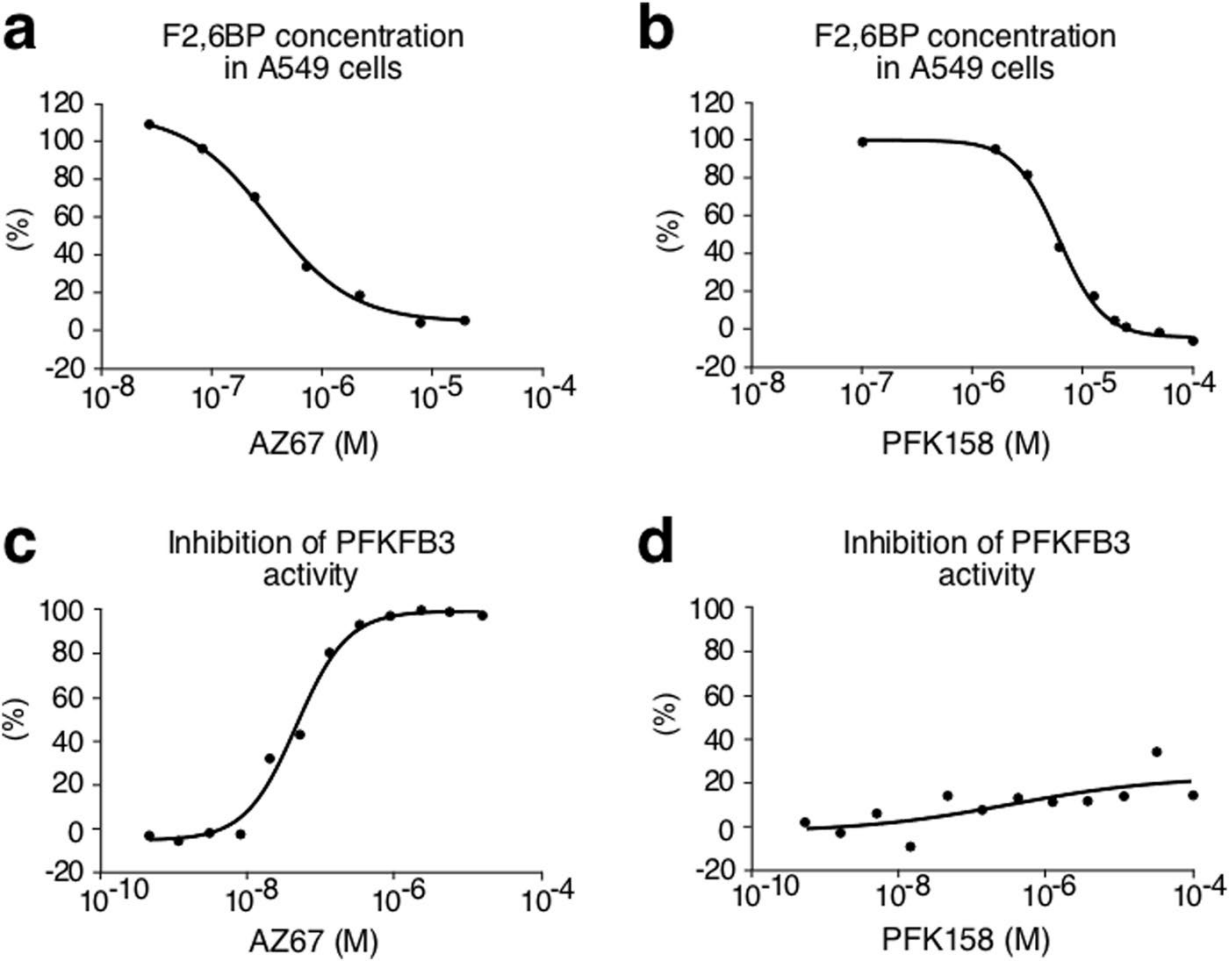
*In vitro* characterization of two PFKFB3 inhibitors. **(a)** Incubation of A549 cells with AZ67 (1 h) dose-dependently decreases F2,6BP concentration. **(b)** Incubation of A549 cells with PFK158 (1 h) dose-dependently decreases F2,6BP concentration. **(c)** Incubation of human recombinant PFKFB3 with AZ67 dose-dependently inhibits PFKFB3 kinase activity. **(d)** Incubation of human recombinant PFKFB3 with PFK158 does not inhibit PFKFB3 kinase activity. See also Supplementary Data 1.

First, we evaluated the efficacy of two known PFKFB3 inhibitors at inhibiting the ability of A549 cells to produce F2,6BP, namely AZ67 (ref.^18^), and PFK158, an improved derivative of the widely used compound, 3-(3-pyridinyl)-1-(4-pyridinyl)-2-propen-1-one 1 (3PO)^19^. As shown in Fig. 1a,b, both compounds (AZ67 and PFK158) were able to reduce the cellular levels of F2,6BP in a dose-dependent manner, with IC50 of 0.51 μM and 5.90 μM, respectively. Next, we investigated whether the reduction of cellular F2,6BP levels was a result of direct PFKFB3 inhibition. To do so, we used an enzymatic cell-free assay, which revealed that AZ67 inhibited the enzymatic activity of PFKFB3 with an IC50 of 0.018 µM (Fig. 1c), a value that is in accordance with previously published results^18^. However, surprisingly, PFK158 had no effect on PFKFB3 enzymatic activity at any of the concentrations tested (up to 100 µM) (Fig. 1d). Accordingly, although PFK158 is able to decrease F2,6BP (Fig. 1b) and glycolytic flux^20^, our data show that these effects are not due to PFKFB3 enzymatic inhibition. Since in this study we are focused specifically on PFKFB3 given its particular protein stability feature and potential impact on neurodegeneration, we did not consider PFK158 for further analyses.

### AZ67 protects neurons against proteasome inhibition and ß-amyloid treatment

Since, in neurons, PFKFB3 is continuously degraded by the proteasome^7^, we reasoned that the stabilization of PFKFB3 caused by proteasomal inhibition may trigger neuronal apoptosis. As shown in Supplementary Fig. S1a, AZ67 lacks toxicity in the range 0.01–100 nM for 24 h in mouse cortical primary neurons. Incubation of neurons with MG132, a widely used proteasomal inhibitor, significantly increased neuronal apoptosis (Fig. S1a), an effect that was dose-dependently counteracted by AZ67 (minimum effective dose, 1 nM; maximum effect at 10 nM), suggesting the involvement of PFKFB3 activity in MG132-mediated neuronal death. To investigate if AZ67 protects neurons from the toxicity caused by PFKFB3 stabilization upon a different kind of stimulus, we next used the amyloidogenic fragment 25–35 of the amyloid-ß peptide (Aβ_25–35_), known to activate glutamate receptors^21^ and to inhibit Cdh1 (ref.^22^), i.e. conditions that stabilize PFKFB3 (ref.^13^). Incubation of neurons with Aß_**25–35**_ increased neuronal apoptosis (Fig. S1a), and this effect was efficiently counteracted by AZ67 in a dose-dependent manner (minimum effective dose, 1 nM; maximum effect at 10 nM), thus suggesting that the excitotoxic effect of Aß_**25–35**_ can, at least in part, be explained by PFKFB3 activation.

### AZ67 prevents glycolytic activation and redox stress upon excitotoxic stimuli in primary neurons

**Figure 2:**
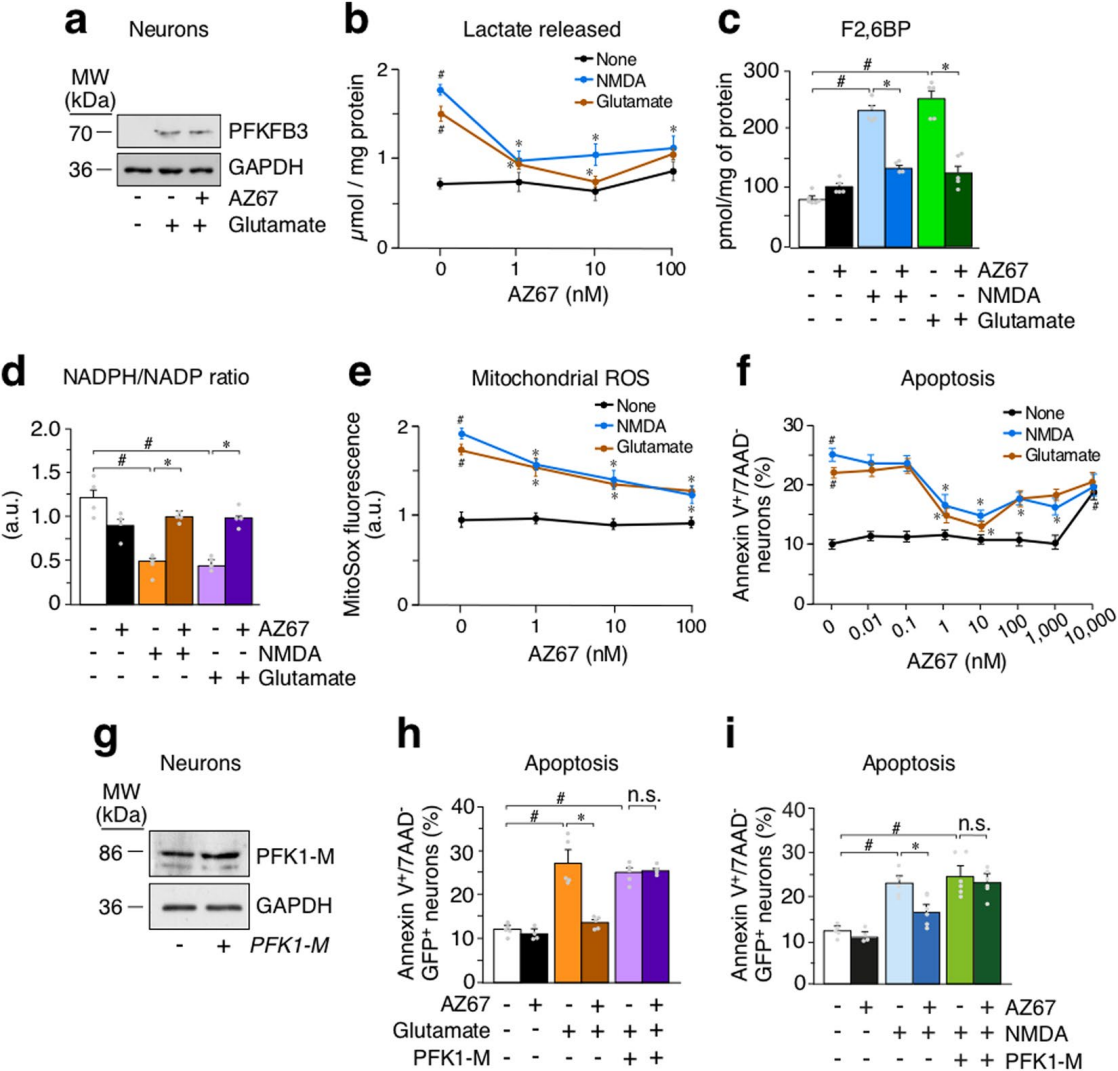
AZ67 prevents glycolytic activation, redox stress and neuronal death upon excitotoxic stimuli in primary neurons. **(a)** Western immunoblotting showing that the treatment of neurons with glutamate (100 µM; 10 mins), followed by washout, stabilized PFKFB3 protein levels after 24 h of incubation. Incubation of neurons with AZ67 (10 nM) for 24 h, after glutamate was removed, did not alter PFKFB3 protein levels. **(b)** Incubation of neurons with AZ67 for 24 h revealed no effect on lactate release. Treatment of neurons with NMDA (100 µM; 10 mins) or glutamate (100 µM; 10 mins), followed by washout, increased lactate released after 24 h of incubation (compare NMDA or glutamate *versus* none values at 0 nM AZ67). Incubation of neurons with AZ67 for 24 h, after NMDA or glutamate was removed, dose-dependently prevented the increase in lactate release. **(c)** Incubation of neurons with AZ67 for 24 h revealed no effect on F2,6BP concentrations. Treatment of neurons with NMDA (100 µM; 10 mins) or glutamate (100 µM; 10 mins), followed by washout, increased F2,6BP after 24 h of incubation. Incubation of neurons with AZ67 (10 nM) for 24 h, after NMDA or glutamate was removed, prevented the increase in lactate release. **(d)** Incubation of neurons with AZ67 for 24 h revealed no significant effect on the NADPH/NADP ratio. Treatment of neurons with NMDA (100 µM; 10 mins) or glutamate (100 µM; 10 mins), followed by washout, decreased the NADPH/NADP ratio after 24 h of incubation. Incubation of neurons with AZ67 (10 nM) for 24 h, after NMDA or glutamate was removed, prevented the decreased NADPH/NADP ratio. **(e)** Incubation of neurons with AZ67 for 24 h revealed no effect on mitochondrial ROS. Treatment of neurons with NMDA (100 µM; 10 mins) or glutamate (100 µM; 10 mins), followed by washout, increased mitochondrial ROS after 24 h of incubation (compare NMDA or glutamate *versus* none values at 0 nM AZ67). Incubation of neurons with AZ67 for 24 h, after NMDA or glutamate was removed, dose-dependently prevented the increase in mitochondrial ROS. **(f)** Treatment of neurons with NMDA (100 µM; 10 mins) or glutamate (100 µM; 10 mins), followed by washout, triggered apoptotic death after 24 h of incubation (compare NMDA or glutamate *versus* none values at 0 nM AZ67). Incubation of neurons with AZ67 for 24 h, after NMDA or glutamate was removed, dose-dependently prevented apoptotic death up to 10 nM, AZ67; at concentrations of 100 nM to 10 µM, AZ67 showed progressive loss of protection. **(g)** Transfection of primary neurons with *Pfk1-M* (4 µg of DNA plasmid) efficiently increased PFK1-M protein levels. **(h)** Treatment of neurons with glutamate (100 µM; 10 mins), followed by washout, triggered apoptotic death after 24 h of incubation. Incubation of neurons with AZ67 (10 nM) for 24 h, after glutamate was removed, prevented apoptotic death. However, AZ67-mediated protection of apoptotic death was abolished when neurons were previously transfected with the full-length cDNA coding for PFK1-M. Apoptosis was analysed only in the efficiently-transfected, GFP^+^ neurons. **(i)** Treatment of neurons with NMDA (100 µM; 10 mins), followed by washout, triggered apoptotic death after 24 h of incubation. Incubation of neurons with AZ67 (10 nM) for 24 h, after NMDA was removed, prevented apoptotic death. However, AZ67-mediated protection of apoptotic death was abolished when neurons were previously transfected with the full-length cDNA coding for PFK1-M. Apoptosis was analysed only in the efficiently-transfected, GFP^+^ neurons. In all cases, data are mean ± S.E.M. values for n = 3 independent culture preparations. #p < 0.05 *versus* none at 0 nM AZ67; *p < 0.05 *versus* the corresponding treatment at 0 nM AZ67 (ANOVA followed by the least significant difference multiple range test). See also Supplementary Data 1 and Statistics Table 1.

To test the ability of AZ67 to protect against the damage caused by an excitotoxic stimuli, neurons were subjected to a short-term incubation with glutamate (100 µM for 10 minutes) followed by a 24 h incubation in glutamate-free culture medium, a widely-used excitotoxic protocol^23^. In good agreement with our previous observations^13^, this treatment triggered PFKFB3 protein stabilization (Figs 2a and S1b) and activated glycolysis, as judged by the increase in the release of the glycolytic end-product, lactate, to the culture medium after 24 h of incubation (Fig. 2b). Interestingly, the increased release of lactate was mimicked by treating neurons with the selective glutamate receptor agonist, N-methyl-D-aspartate (NMDA) (Fig. 2b), and was dose-dependently abrogated by incubation of neurons with AZ67 immediately after the excitotoxic stimuli during 24 h (Fig. 2b). The minimum concentration of AZ67 that showed to be fully efficient at preventing the increase in lactate release was 1 nM, although at 10 nM, AZ67 was maximally effective in the glutamate-mediated stimulus (Fig. 2b). In good consistency with PFKFB3 protein stabilization^13^ (Fig. 2a), treatment of neurons with the excitotoxic stimuli increased the levels of the PFKFB3 product, F2,6BP, by almost ~3-fold (Fig. 2c), indicating increased PFKFB3 enzymatic activity. Notably, the increased F2,6BP levels were fully abolished by AZ67 at 10 nM (Fig. 2c) without affecting PFKFB3 protein stability (Fig. 2a). These data indicate that the pharmacological inhibition of PFKFB3 activity in neurons is sufficient to prevent excitotoxic stimuli-mediated activation of glycolysis. In contrast to neurons, astrocytes normally express high levels of PFKFB3 that are responsible for the high glycolytic phenotype of these glial cells^4,7^. Moreover, by activating PFKFB3, glycolysis further increases in astrocytes upon inhibition of mitochondrial cytochrome *c* oxidase with nitric oxide^3,4^. However, AZ67, at the low dose (10 nM) that is able to inhibit PFKFB3 activity in neurons (Fig. 2b), did not inhibit basal or nitric oxide-stimulated glycolysis in astrocytes (Fig. S1c). Since an increase in glycolysis leads to the impairment in the ability of neurons to regenerate NADPH through PPP activity^7^, we next assessed the redox state of this cofactor. As shown in Fig. 2d, stimulation of glutamate receptors triggered NADPH oxidation, a hallmark of PPP inhibition^7,24^, as judged by the decreased NADPH/NADP ratio, an effect that was abrogated by incubating neurons with AZ67 (10 nM). Given that PPP-mediated regeneration of oxidized NADPH is essential for preventing the redox stress in neurons^7,13^ that accompanies mitochondrial damage in several neurodegenerative diseases^25,26^, we next investigated mitochondrial reactive oxygen species (ROS). In good agreement with this notion, treatment of neurons with the excitotoxic stimuli promoted an increase in mitochondrial ROS (Fig. 2e), and this effect was abolished by AZ67 (10 nM) (Fig. 2e). Thus, inhibition of PFKFB3 activity upon an excitotoxic stimuli prevents the aberrant activation of glycolysis in neurons that leads to redox stress.

### The neuroprotective effect of AZ67 against excitotoxicity is lost by genetically hampering glycolytic inhibition

Next, we aimed to further confirm whether AZ67, by preventing the activation of glycolysis in neurons, could account for the neuronal death associated with the excitotoxic stimuli. To do so, neurons were incubated with glutamate or NMDA, as above, and apoptosis assessed by annexin V^+^/7AAD^−^ staining using flow cytometry. As shown in Fig. 2f, both types of excitotoxic stimuli significantly increased apoptotic neuronal death. Notably, this effect was dose-dependently prevented by AZ67, being 1 nM the minimum effective concentration and 10 nM the maximum dose showing protection (Fig. 2f). A progressive loss of protection was observed at AZ67 concentrations ≥100 nM (Fig. 2f). To address whether the neuronal protection exerted by AZ67 was a consequence of preventing the glycolytic activation, we assessed whether overexpression of the glycolytic enzyme, PFK1-muscle isoform, was able to rescue AZ67-mediated neuroprotection. We focused on the muscle PFK1 isoform (PFK1-M) given its very low sensitivity to F2,6BP allosteric activation^27^ and, hence, its independence on PFKFB3 levels to fully activate glycolysis^28^. Accordingly, neurons were first transfected with the full-length cDNA encoding for PFK1-M (Figs 2g and S1d), and then subjected to the excitotoxic insults. As shown in Fig. 2h,i, PFK1-M over-expression was able to abrogate the neuroprotection caused by AZ67 (10 nM) against glutamate or NMDA-mediated neuronal apoptosis. These results confirm that the neuroprotection exerted by AZ67 is a consequence of its ability to prevent glycolytic activation.

### AZ67 prevents the metabolic switch from PPP to glycolysis, redox and mitochondrial stress, and apoptosis in an *in vitro* model of ischemia/reperfusion in primary neurons

**Figure 3:**
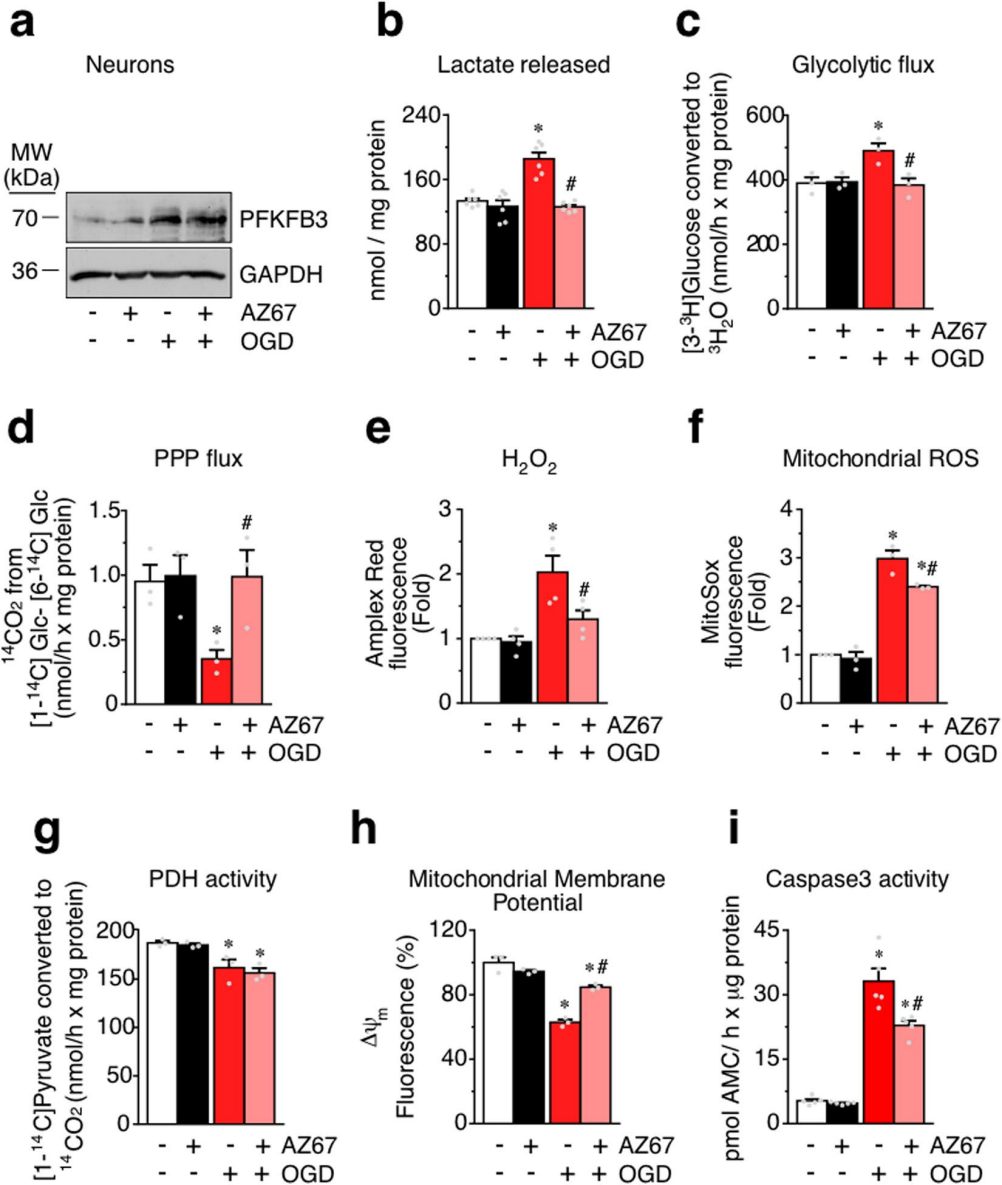
AZ67 prevents the metabolic switch from PPP to glycolysis, redox and mitochondrial stress, and apoptosis in an *in vitro* model of ischemia/reperfusion in primary neurons. **(a)** Western immunoblotting showing that the treatment of neurons with OGD (3 h) followed by reoxygenation (plus glucose) (4 h), stabilized PFKFB3 protein levels. Incubation of neurons with AZ67 (10 nM) during the 4 h of reoxygenation did not alter PFKFB3 protein levels. **(b)** Incubation of neurons with AZ67 for 4 h revealed no effect on lactate release. Treatment of neurons with OGD (3 h) followed by reoxygenation (plus glucose) (4 h) increased lactate released. Incubation of neurons with AZ67 (10 nM) during the 4 h of reoxygenation prevented the increase in lactate release. **(c)** Incubation of neurons with AZ67 for 4 h revealed no effect on the glycolytic flux, as assessed by the formation of ^3^H_2_O from [3-^3^H]glucose. However, treatment of neurons with OGD (3 h) followed by reoxygenation (plus glucose) (4 h) increased the glycolytic flux. Incubation of neurons with AZ67 (10 nM) during the 4 h of reoxygenation prevented the increase in the glycolytic flux. **(d)** Incubation of neurons with AZ67 for 4 h revealed no effect on the pentose-phosphate pathway (PPP) flux, as assessed by the difference in the formation of ^14^CO_2_ from [1-^14^C]- and from [6-^14^C]glucose. However, treatment of neurons with OGD (3 h) followed by reoxygenation (plus glucose) (4 h) decreased the PPP flux. Incubation of neurons with AZ67 (10 nM) during the 4 h of reoxygenation prevented the decrease in the PPP flux. **(e)** Incubation of neurons with AZ67 for 4 h revealed no effect on H_2_O_2_ release, as assessed by the fluorescence of AmplexRed. However, treatment of neurons with OGD (3 h) followed by reoxygenation (plus glucose) (4 h) increased H_2_O_2_ release. Incubation of neurons with AZ67 (10 nM) during the 4 h of reoxygenation prevented the increase in H_2_O_2_ release. **(f)** Incubation of neurons with AZ67 for 4 h revealed no effect on mitochondrial ROS formation, as assessed by MitoSox fluorescence by flow cytometry. However, treatment of neurons with OGD (3 h) followed by reoxygenation (plus glucose) (4 h) increased mitochondrial ROS. Incubation of neurons with AZ67 (10 nM) during the 4 h of reoxygenation significantly prevented the increase in mitochondrial ROS formation. **(g)** Incubation of neurons with AZ67 for 4 h revealed no effect on pyruvate dehydrogenase (PDH) activity, as assessed by the conversion of [1-^14^C]pyruvate in ^14^CO_2_. However, treatment of neurons with OGD (3 h) followed by reoxygenation (plus glucose) (4 h) decreased PDH activity that was not altered by incubation of neurons with AZ67 (10 nM) during the 4 h of reoxygenation. **(h)** Incubation of neurons with AZ67 for 4 h revealed no effect on mitochondrial membrane potential (∆ψ_m_), as assessed by flow cytometry. However, treatment of neurons with OGD (3 h) followed by reoxygenation (plus glucose) (4 h) decreased ∆ψ_m_. Incubation of neurons with AZ67 (10 nM) during the 4 h of reoxygenation prevented the decreased ∆ψ_m_. **(i)** Incubation of neurons with AZ67 for 4 h revealed no effect on caspase-3 activity, a measure of apoptosis. However, treatment of neurons with OGD (3 h) followed by reoxygenation (plus glucose) (4 h) increased apoptosis. Incubation of neurons with AZ67 (10 nM) during the 4 h of reoxygenation prevented the increase in apoptosis. In all cases, data are mean ± S.E.M. values for n = 3 independent culture preparations. #p < 0.05 *versus* OGD at 0 nM AZ67; *p < 0.05 *versus* the corresponding normoxic condition (ANOVA followed by the least significant difference multiple range test). See also Supplementary Data 1 and Statistics Table 1.

Next, we aimed to elucidate whether AZ67 is able to prevent the metabolic switch and loss of survival triggered by a different type of excitotoxic stimulus in neurons. Given that neuronal damage associated with the loss of oxygen and nutrient supply is known to take place through an excitotoxic pathway^29,30^, we prompted to investigate the potential beneficial effect of AZ67 in the *in vitro* model of oxygen and glucose deprivation (OGD)^31^. To do so, primary neurons were subjected to a 3 hours OGD incubation followed by a 4 hours reoxygenation (plus glucose) incubation period, a characterized *in vitro* model of ischemia^31^. In good consonance with the excitotoxic models, OGD/reoxygenation triggered PFKFB3 protein stabilization (Figs 3a and S1e) and activated glycolysis, as judged by the increase in the release of lactate to the culture medium at the end of the reoxygenation period (Fig. 3b). Interestingly, the increased release of lactate was abrogated by incubation of neurons with AZ67 (10 nM) immediately after OGD, during the reoxygenation period (Fig. 3b). Notably, the increased release of lactate was fully abolished by AZ67 (10 nM) (Fig. 3b) without affecting PFKFB3 protein stability (Fig. 3a). To further confirm the activation of glycolysis in the OGD/reperfusion model, we determined the rate of [3-^3^H]glucose conversion into ^3^H_2_O, a *bona fide* measure of the glycolytic flux^7,32^. Treatment of neurons with the OGD/reoxygenation protocol increased the glycolytic flux (Fig. 3c). Since increased glycolysis impairs glucose metabolism through PPP in neurons^7^, we next assessed the rate of PPP flux using a radiometric approach based on determining the difference in the release of ^14^CO_2_ from neurons incubated with either [1-^14^C]glucose or [6-^14^C]glucose^7^. As shown in Fig. 3d, OGD/reoxygenation triggered the inhibition of PPP flux. Interestingly, both the increase in glycolysis and the decrease in PPP were abrogated by incubating neurons with AZ67 (10 nM) (Fig. 3c,d). Since PPP is essential to maintain the antioxidant status of neurons^7,13^, we investigated ROS. Treatment of neurons with the OGD/reoxygenation protocol increased H_2_O_2_ release from neurons (Fig. 3e) and mitochondrial ROS (Fig. 3f), effects that were significantly attenuated by AZ67 (10 nM) (Fig. 3e,f). These data indicate that OGD/reperfusion triggers a metabolic switch from PPP to glycolysis that is associated with redox stress, and that these effects are significantly prevented by AZ67. As expected, OGD/reoxygenation impaired mitochondrial intermediary metabolism as observed by the decreased oxidation of [1-^14^C]pyruvate (Fig. 3g), a measure of pyruvate dehydrogenase (PDH) activity. However, PDH activity was unaffected by AZ67, neither when added alone or in the OGD/reoxygenation model (Fig. 3g). Altogether, these data strongly suggest that AZ67 primarily affects glucose transformation through glycolysis and, indirectly, PPP, without altering mitochondrial intermediary metabolism. Furthermore, OGD/reperfusion triggered a loss in the mitochondrial inner membrane potential (∆ψ_m_) (Fig. 3h), likely consequence of the associated redox stress-mediated damage of the mitochondrial respiratory chain^15–17^. Interestingly, in good agreement with the redox stress rescue exerted by AZ67 (Fig. 3e,f), this compound significantly prevented the ∆ψ_m_ loss caused by OGD/reperfusion (Fig. 3h). To ascertain if AZ67 affected neuronal survival in the OGD/reperfusion model, we assessed caspase-3 activity as a measure of apoptosis. As shown in Fig. 3i, OGD/reperfusion increased neuronal apoptosis, an effect that was significantly rescued by AZ67 (10 nM).

### *In vivo* AZ67 administration protects mice against the motor discoordination caused by a brain ischemia/reperfusion model

**Figure 4:**
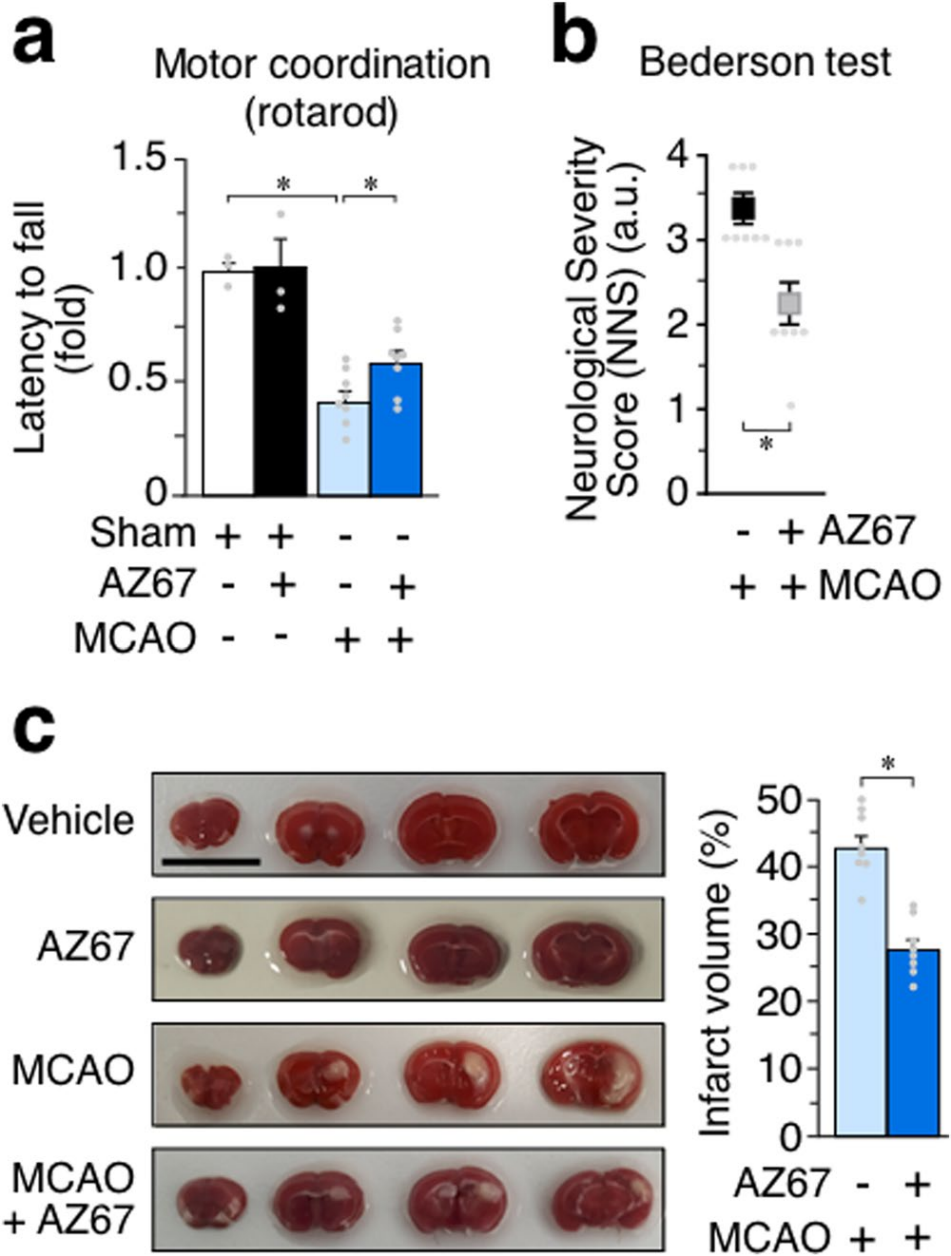
*In vivo* AZ67 administration protects mice against neurological impairment, motor discoordination and brain injury caused by a brain ischemia/reperfusion model. **(a)** Motor coordination, analysed 24 h after a transient MCAO episode in mice, revealed a ~40% performance (rotarod) when compared with the sham-operated animals (100% performance). This effect was significantly prevented by the intravenous administration of AZ67 (60 mg/kg of body weight) immediately after the ischemic episode (~60% performance). **(b)** Neurological Severity Score (NNS), examined 24 h after a transient MCAO episode in mice, revealed severe neurological deficit according to the Bederson test when compared with the sham-operated animals. This effect was significantly prevented by the intravenous administration of AZ67 (60 mg/kg of body weight) immediately after the ischemic episode. (NNS = 0 both for vehicle or AZ67 in the sham-operated animals). **(c)** Infarcted brain volume, analysed 24 h after a transient MCAO episode in mice, was ~43% of the brain of the sham-operated animals. This effect was significantly prevented by the intravenous administration of AZ67 (60 mg/kg of body weight) immediately after the ischemic episode (~27% of infarcted volume). Left panel shows pictures of the brain sections of a representative animal for each experimental group. Bar, 1 cm. In all cases, data are mean ± S.E.M. values for 8 male mice. *p < 0.05 (ANOVA followed by the least significant difference multiple range test). See also Supplementary Data 1 and Statistics Table 1.

Finally, we aimed to investigate if AZ67 was able to exert neuroprotection *in vivo*. Since it is very well documented that brain injury in stroke occurs through an excitotoxic mechanism^29,30^, we studied whether AZ67 protected against damage caused in a mouse model of stroke. To achieve this, we induced a transient ischemia (30 min) by occlusion of the middle carotid artery (MCAO model), followed by 24 h reperfusion as described by a well-established protocol^31,33^. Twenty-four hours after the transient MCAO episode, mice were subjected to the rotarod test, which revealed a ~40% performance of motor coordination in the MCAO group when compared with the sham-operated animals treated with vehicle (Fig. 4a). Following this analysis, animals were then subjected to neurological examination following the Bederson test^34^. As shown in Fig. 4b, the results revealed severe neurological impairment (score >3) by MCAO. Immediately after the Bederson test, mice were euthanized to determine the percentage of infarcted volume in the brain, which resulted to be ~43% in the MCAO group (0% in the vehicle, sham-operated animals) (Fig. 4c). AZ67 (60 mg/kg of body weight), or vehicle, were administered intravenously through the jugular vein immediately after the ischemic episode, at the start of the reperfusion. AZ67 administration in the sham-operated animals showed no signs of neurological deficit (NNS = 0 both for vehicle and AZ67; Fig. 4a), motor discoordination (Fig. 4b) or brain injury (Fig. 4c) after 24 h when compared with vehicle, sham-operated mice, indicating lack of AZ67 toxicity at the dose used. Although the use of only one dose of AZ67 might represent a study limitation, notably, AZ67 administration at the selected dose of 60 mg/kg of body weight significantly improved the neurological deficit (NNS = ~2) (Fig. 4a), prevented the motor discoordination (~60% performance) (Fig. 4b) and decreased the infarcted brain volume to 27% (Fig. 4c).

## Discussion

Here we show that pharmacological inhibition of PFKFB3 activity, by preventing glycolytic activation, protects neurons against excitotoxicity both in the NMDA and glutamate receptor activation models and in the oxygen-glucose deprivation/reperfusion model. Furthermore, PFKFB3 inhibition also showed brain damage protection in the MCAO ischemic/reperfusion model *in vivo*. To our knowledge, this is the first time showing that inhibition of glycolysis, by the use of a small-molecule compound, shows a beneficial effect in a neurological disease model. In cancer cells, several PFKFB3 inhibitors of different chemical classes have been reported to inhibit glycolysis, on which these cells rely for proliferation and survival^35–39^. Amongst these PFKFB3 inhibitors, 3PO and its derivative PFK158 have been reported to reduce the cellular levels of F2,6BP, inhibit glucose uptake and lactate production, thus facilitating apoptosis in cancer cells. However, on our hands, PFK158 is inactive as PFKFB3 inhibitor in a purified human recombinant PFKFB3 enzymatic assay, at least at concentrations up to 100 μM. Intriguingly, similar results were previously reported for 3PO^18,39^. Whether these compounds inhibit glycolysis by interfering with a glycolytic target different to PFKFB3 remains to be elucidated. In contrast, AZ67 is a *bona-fide* PFKFB3 inhibitor^18^ that, on our conditions, inhibited human recombinant PFKFB3 kinase activity at with an IC50 of 18 nM and decreased cellular F2,6BP production in A549 cells with an IC50 of 510 nM. When compared with astrocytes and other tissues, neurons are, by far, the type of cell showing the smallest PFKFB3 abundance, which is virtually absent^7^. In good agreement with this, we found that lower concentrations of AZ67 (in the 1–10 nM range) were sufficient to efficiently abrogate the enhancements in F2,6BP, glycolysis and apoptosis in neurons. However, AZ67 was unable to decrease basal glycolysis nor to rescue the increased glycolytic activation in astrocytes. *In vivo* administration of the PFKFB3 inhibitor in the MCAO model could, in principle, enter all cells of the organism to broadly inhibit PFFKFB3 activity. However, our data showing lack of effect of AZ67 in astrocytic glycolysis strongly supports the notion that neuronal glycolysis would be more sensitive than astrocytic to AZ67, which is in good agreement with the large difference in PFKFB3 abundance between neurons and astrocytes^7^. This feature can be advantageous when determining the specific administration doses for future pre-clinical trials.

The rationale for targeting PFKFB3 and glycolysis as a therapeutic strategy for neurodegeneration relies on the physiological regulation of PFKFB3 protein stability in neurons. Thus, PFKFB3 is continuously degraded by the proteasome to keep glycolysis low in healthy neurons; however, PFKFB3 becomes stabilized upon proteasomal inhibition, leading to increased glycolysis and decreased PPP, which ultimately causes redox stress and neuronal death^7^. In good agreement with this notion, our data show that PFKFB3 inhibition by AZ67 exerts neuroprotection after blocking the proteasome with MG132. Furthermore, AZ67 prevented the neuronal death triggered by the Alzheimer’s disease-related peptide Aß_**25–35**_, known to promote the degradation of Cdh1, i.e. the APC/C-cofactor necessary for PFKFB3 ubiquitylation and proteasomal degradation^22^. In the neuronal models of excitotoxicity and OGD/reoxygenation, the increase in glycolysis was paralleled with NADPH oxidation and PPP inhibition, respectively. NADPH oxidation impairs glutathione regeneration thus causing redox stress^7,24^, a feature that we have herein confirmed according to the increase mitochondrial ROS upon the excitotoxic and OGD/reoxygenation insults. Interestingly, AZ67 was able to rescue the enhancement in glycolysis, PPP inhibition, NADPH oxidation and redox stress, supporting the notion that restoring the equilibrium between glycolysis and PPP is a suitable and efficient neuroprotective strategy. Importantly, the neuroprotection exerted by AZ67 was abolished by over-expressing PFK1-M, an enzyme that activates glycolysis independently on F2,6BP levels^27^. This result demonstrates that the neuroprotective effect of AZ67 is due to its ability to inhibit the increase in glycolysis.

Excitotoxicity is a hallmark of various neurodegenerative diseases including stroke. Moreover, there is both pre-clinical^40^ and clinical^41^ evidence that tissue plasminogen activator (tPA), the only approved drug treatment for acute ischemic stroke, despite its obvious benefits presents adverse effects in the brain, including excitotoxicity potentiation^42^. Although neuroprotective strategies have been focused on NMDA receptor antagonists, unfortunately none of them have been successful, likely because functional NMDA receptors are essential for the normal brain physiology. Our data showing that targeting downstream the NMDA receptors activation -such as neuronal glycolysis-, rather than the NMDA receptors themselves, shows neuroprotection strongly suggest that targeting PFKFB3 would be a suitable alternative strategy for the prevention of the deleterious effects of NMDA receptor overstimulation in stroke and related excitotoxicity-associated neurological disorders, such as, amongst others, traumatic brain injury, Alzheimer’ or Parkinson’ diseases.

## Methods

### Ethical use of animals

All animal procedures we performed according to the European Union Directive 86/609/EEC and Recommendation 2007/526/EC, regarding the protection of animals used for experimental and other scientific purposes, enforced in Spanish legislation under the directive RD1201/2005. All protocols were approved by the Bioethics Committee of the University of Salamanca and by the Junta de Castilla y Leon (registration number 080).

### PFKFB3 Enzymatic assay

Recombinant full length human PFKFB3 protein purified from Sf9 baculoviral system acquired from SignalChem (Cat. #P323-30G). ATP, fructose-6-phosphate (F6P and other chemicals were from Sigma-Aldrich. ADP detection system (ADP-Glo) was purchased from Promega. Inhibitors were synthesized as described by Boyd *et al*.^18^. The kinase activity of the PFKFB3 protein was detected by measuring production of ADP from ATP in the presence of F6P. The reactions were assembled in 384 well plates in a total volume of 25 µl. Test compounds were serially diluted in dimethyl sulfoxide (DMSO). Reactions were set up by mixing test compounds with the enzyme and pre-incubating for 15 min. ATP and F6P were next added to initiate the reactions. The final assay composition included: 100 mM Tris-HCl pH 8.0, 4 mM MgCl_2_, 5 mM KH_2_PO_4_, 5 mM DTT, 20 mM KF, 0.02% BSA, 1% DMSO (from the compounds), 15 nM enzyme, 20 µM ATP (Km = 16 µM) and 10 µM F6P (Km = 6 µM). We used a low concentration of PFKFB3 protein (15 nM) since concentrations higher than 50 nM showed to hydrolyze ATP in the absence of F6P. Thus, under the conditions used in our experiments, ADP formation was solely due to F6P phosphorylation by PFKFB3. The kinase reactions were allowed to proceed for 1 hour at room temperature. Aliquots of the reaction mixtures (5 µl) were transferred to fresh white 384 well plates and mixed with 5 µl of the ADP-Glo reagent, followed by incubation for 30 min. The luminescent kinase detection reagent was added (10 µl) and, following additional incubation for 15 min, the plates were read with a luminescence plate reader (Analyst HT). Positive (no enzyme; 100% inhibition) and negative (DMSO instead of AZ67 or PFK158; 0% inhibition) control samples were assembled in each assay plate and were used to calculate percent inhibition values of test compounds.

### Fructose-2,6-bisphosphate determinations

For F2,6BP determinations, cells were lysed in 0.1 N NaOH and centrifuged (20,000 × *g*, 20 min). An aliquot of the homogenate was used for protein determination, and the remaining sample was heated at 80 °C (5 min), centrifuged (20.000 × *g*, 20 min) and the resulting supernatant used for the determination of F2,6BP concentrations using the coupled enzymatic reaction and F2,6BP standards as described by Van Schaftingen^43^.

### Cell culture

Primary cultures of C57BL/6J mice cortical neurons were prepared from foetal animals of 14.5 days of gestation, seeded at 1.8·10^5^ cells/cm^2^ in plastic plates coated with poly-D-lysine (10 mg/ml) and incubated in Neurobasal (Life Technologies) supplemented with 2 mM glutamine, 5 mM of glucose, 0.25 mM pyruvate and 2% B27 supplement (Life Technologies). Cells were incubated at 37 °C in a humidified 5% CO_2_-containing atmosphere. At 72 hours after plating, medium was replaced using Neurobasal (Life Technologies) supplemented with 2 mM glutamine, 5 mM glucose, 0.25 mM pyruvate and 2% B27 supplement (Life Technologies) minus antioxidants (MAO; i.e., lacking vitamin E, vitamin E acetate, superoxide dismutase, catalase and glutathione). Six days after plating medium was replaced again. Cells were used at day 9. Primary cultures of brain cortical astrocytes were prepared from either C57BL/6 mice of 0–1 days-old neonates. Cell suspensions were seeded at 2.5 × 10^5^ cells in 175 cm^2^ plastic flasks and incubated in Dulbecco’s Modified Eagle’s Medium (DMEM) supplemented with 10% fetal bovine serum (BSA). Non-astroglial cells were detached after 7 DIV by shaking the flasks at 200 r.p.m. overnight and discarding the supernatant. The remaining attached, astrocyte-enriched cells were re-seeded in different size plastic plates and further incubated for 7 DIV for the experiments. Adenocarcinomic human alveolar basal epithelial cells (A549 cells) were seeded at 10^4^ cells/cm^2^ in Dulbecco’s modified Earls Medium (DMEM; Sigma-Aldrich) supplemented with 10% fetal calf serum (FCS).

### Cell transfections

Primary neurons were transfected with 1.6 µg/mL of a pIRES2-EGFP plasmid vector (Invitrogen) harbouring the full-length cDNA coding for the human muscle 6-phosphofructo-1-kinase muscle isoform (PFK1-M)^28^ (accession number, NM_000289.1) using Lipofectamine LTX-PLUS Reagent (Life Technologies) according with manufacturer’s protocol. Transfections were performed 24 hours before cells collection. Control cells were transfected with the empty vector.

### Cell treatments

For NMDA receptors activation, neurons at 8 days *in vitro* were incubated with 100 *μ*M glutamate (plus 10 *μ*M glycine) or 100 *μ*M NMDA (plus 10 *μ*M glycine) for 10 minutes. Neurons were then washed and further incubated in culture medium with the PFKFB3 inhibitors for 24 hours. For amyloid-ß treatment, the active truncated amyloid-β peptide Aβ_25–35_ (BioNova Cientifica S.L., Madrid, Spain) was used. Aß_25–35_ was dissolved in distilled water at a concentration of 1 mg/ml and then incubated at 37 °C for 3 days to induce its oligomerization^44^. We have shown that Aß_25–35_ exerts neurotoxicity following identical mechanism to the full-length Aβ_1–42_ peptide^21^. Neurons were incubated in culture medium containing oligomerized Aβ_25–35_ (10 µM) or the corresponding scramble non-aggregable peptide (Aβ_35–25_) (BioNova Cientifica S.L.), which was used as control. Neurons at 8 days *in vitro* were incubated with Aß_25–35_ plus the PFKFB3 inhibitors for 24 hours. To inhibit the proteasome, neurons were incubated with MG132 (10 µM) for 2 hours. To inhibit cytochrome c oxidase activity, astrocytes at 14 days *in vitro* were incubated with the nitric oxide donor, DETA-NONOate (0.5 mM)^4^ for 4 hours.

### Oxygen and glucose deprivation (OGD)/reoxygenation protocol

After 8 days in culture, neurons were subjected to oxygen and glucose deprivation (OGD) by incubating cells at 37 °C in an incubator equipped with an air lock and continuously gassed with 95% N_2_/5% CO_2_, for 3 hours. The incubation medium (Neurobasal medium without glucose) was previously gassed with 95% N_2_/5% CO_2_ for 5 min. In parallel, neurons were incubated in Neurobasal complete medium (normoxia condition) at 37 °C in a humidified atmosphere of 95% air/5% CO_2_. After OGD, neurons were further incubated for 4 hours with (or not) AZ67 10 nM in Neurobasal medium at 37 °C in a humidified atmosphere of 95% air/5% CO_2_ (reoxygenation after OGD)^31^.

### Western blotting

Neurons were lysed in RIPA buffer (2% sodium dodecylsulphate, 2 mM EDTA, 2 mM EGTA and 50 mM Tris pH 7.5), supplemented with protease and phosphatase inhibitor cocktail (100 μM phenylmethylsulfonyl fluoride, 50 μg/ml antipapain, 50 μg/ml pepstatin, 50 μg/ml amastatin, 50 μg/ml leupeptin, 50 μg/ml bestatin, 1 mM *o*-vanadate, 50 mM NaF, and 50 μg/ml soybean trypsin inhibitor) and boiled for 5 min. Extracts were centrifuged at 13,000 × *g* for 5 min at 4 °C, and aliquots of lysates (50 μg protein, unless otherwise stated) were subjected to sodium dodecyl sulfate-polyacrylamide (SDS-PAGE) electrophoresis on a 8, 10 or 12% acrylamide gel (MiniProtean, Bio-Rad) including PageRuler Plus Prestained Protein Ladder (Thermo). The resolved proteins were transferred electrophoretically to nitrocellulose membranes (Hybond-ECL, Amersham Bioscience Europe GmbH, Barcelona, Spain). Membranes were blocked with 5% (w/v) low-fat milk in 20 mM Tris, 500 mM NaCl, and 0.1% (w/v) Tween 20, pH 7.5, for 1 h. After blocking, membranes were immunoblotted with primary antibodies at dilutions ranging from 1:500 to 1:40,000 overnight at 4 °C. After incubation with the secondary antibodies (all at 1:10,000 dilution), membranes were immediately incubated with the enhanced chemiluminescence kit WesternBright ECL (Advansta, Menlo Park, California, USA) for 2 min or SuperSignal West Femto Maximum Sensitivity Substrate (Thermo Scientific, Offenbach, Germany) for 5 min, before exposure to Fuji Medical X-Ray film (Fujifilm), and the autoradiograms scanned. Biologically independent replicates were always performed (Supplementary Fig. S5), and a representative western blot is shown.

### Primary antibodies for western blotting

Immunoblotting was performed using mouse monoclonal anti-PFKFB3 (1:500) (H0005209-M08, Novus Biologicals), mouse monoclonal anti- glyceraldehyde dehydrogenase (GAPDH; 1:40,000) (AM4300, Ambion) and antiserum against muscle PFK1 isoform (PFK1-M; 1:10)^4^.

### Secondary antibodies for western blotting

Immunoblotting was performed using horseradish peroxidase-conjugated goat anti-rabbit IgG and goat anti-mouse IgG (Santa Cruz Biotechnologies).

### Mitochondrial ROS

Mitochondrial ROS was detected using the fluorescent probe MitoSox (Life Technologies). Cells were incubated with 2 μM of MitoSox for 30 minutes at 37 °C in a 5% CO_2_ atmosphere in Hank’s Balanced Salt Solution (HBSS buffer); (NaCl 134.2 mM; KCl 5.26 mM; KH_2_PO_4_ 0.43 mM; NaHCO_3_ 4.09 mM; Na_2_HPO_4_·2H_2_O 0.33 mM; glucose 5.44 mM; HEPES 20 mM; CaCl_2_·2H_2_O 4 mM; pH 7.4). Cells were then washed with PBS (phosphate-buffered saline, 0.1 M) and collected by smooth trypsinization. MitoSox fluorescence was assessed by flow cytometry and expressed in arbitrary units (see, also, Supplementary Fig. S2 for the flow cytometry workflow for MitoSox).

### H_2_O_2_ determination

For H_2_O_2_ assessments, AmplexRed (Life Technologies, New York, USA) was used. Cells grown on 96 wells plates were washed with PBS and incubated in KRPG buffer (NaCl 145 mM; Na_2_HPO_4_ 5.7 mM; KCl 4.86 mM; CaCl_2_ 0.54 mM; MgSO_4_ 1.22 mM; glucose 5.5 mM: pH 7.35) in the presence of 9.45 μM AmplexRed containing 0.1 U/ml of horseradish peroxidase. Luminescence was recorded for 2 h at 30 minutes intervals using a Varioskan Flash (Thermo Fisher, Vantaa, Finland) spectrofluorometer (excitation 538 nm; emission 604 nm). Slopes were used for calculations of the rates of H_2_O_2_ formation.

### Flow cytometric analysis of apoptotic cell death

APC-conjugated annexin-V and 7-amino-actinomycin D (7-AAD) (Becton Dickinson Biosciences, BDB, San Jose, CA, USA) were used to determine quantitatively the percentage of apoptotic neurons by flow cytometry. Cells were stained with annexin V-APC and 7-AAD, following the manufacturer’s instructions, and were analysed on a FACScalibur flow cytometer (15 mW argon ion laser tuned at 488 nm; CellQuest software, Becton Dickinson Biosciences) using the CellQuest software (BDB). Both GFP^+^ and GFP^−^ cells were analyzed separately, and the annexin V-APC-stained cells that were 7-AAD-negative were considered to be apoptotic (see, also, Supplementary Fig. S3 for the flow cytometry workflow for apoptosis).

### Active caspase-3 determination

A fluorimetric caspase 3 assay kit (Sigma-Aldrich) was used following the manufacture’s protocol. This assay is based on the hydrolysis of the peptide substrate Ac-DEVD-AMC (acetyl-Asp-Glu-Val-Asp-7-amino-4-methylcoumarin) by caspase-3, which results in the release of fluorescent 7-amino-4-methylcoumarin (AMC). In brief, cells were lysed with 50 mM HEPES, 5 mM CHAPS, 5 mM DTT, pH 7.4 for 20 min on ice, and the assay buffer containing the Ac-DEVD-AMC substrate (20 mM HEPES, 2 mM EDTA, 0.1% CHAPS, 5 mM DTT, 16 µM Ac-DEVD-AMC, pH 7.4) was added. Aliquots of 200 µl were transferred to a 96-wells plate and the fluorescence recorded for 30 mins at 5 mins intervals at 37 °C (λ_exc_ = 360 nm, λ_em_ = 460 nm). CSP-3 activity was determined as AMC release rate extrapolating the slopes to those obtained from an AMC standard curve. Results are expressed as fold change, arbitrarily assigning the value of 1 to control cells.

### NADPH/NADP^+^ ratio determination

This was performed using the colorimetric NADPH/NADP assay kit (Abcam). Cells were re-suspended in 500 µl of NADPH/NADP extraction buffer, vortexed and centrifuged at 14,000 rpm for 5 minutes to remove insoluble material. The supernatant was used for NADPH plus NADP measurement. NADPH was determined in 200 µl of the supernatant, after heated at 60 °C for 30 minutes to decompose NADP. Actual NADP and NADPH concentrations were calculated by extrapolating values to a NADPH standard curve (0–100 pmol/well).

### Determination of the pentose-phosphate pathway (PPP) flux

PPP flux was measured in 8 cm^2^ flasks of adherent cells at 60–70% confluence containing a central microcentrifuge tube with 0.8 ml benzethonium hydroxide (Sigma) for ^14^CO_2_ equilibration. Incubations were carried out in KRPG containing 5.5 mM D-glucose at 37 °C in the air-thermostatized chamber of an orbital shaker (Forma Benchtop Orbital Shaker, Model 420, Thermo Fischer). To ensure adequate oxygen supply for oxidative metabolism throughout the incubation period, flasks were filled with oxygen before being sealed. To measure the carbon flux from glucose through the PPP, cells were incubated in KRPG (5 mM D-glucose) buffer supplemented with 0.5 μCi D-[1-^14^C]glucose or D-[6-^14^C]glucose for 90 min, as previously described^7,32^. Incubations were then terminated by the addition of 0.2 ml 20% perchloric acid (Merck Millipore) for 30 min before the benzethonium hydroxide (containing ^14^CO_2_) was removed, and the radioactivity was measured with a liquid scintillation analyzer (Tri-Carb 4810 TR, PerkinElmer). PPP flux was calculated as the difference between ^14^CO_2_ production from [1-^14^C]glucose (which decarboxylates through the 6-phosphogluconate dehydrogenase–catalyzed reaction) and that of [6-^14^C]glucose (which decarboxylates through the TCA cycle).

### Determination of the glycolytic flux

Glycolytic flux was measured in 8 cm^2^ flasks of adherent cells at 60–70% confluence containing a central microcentrifuge tube with 1 ml H_2_O for ^3^H_2_O equilibration. Incubations were carried out in KRPG containing 5.5 mM D-glucose at 37 °C in the air-thermostatized chamber of an orbital shaker (Forma Benchtop Orbital Shaker, Model 420, Thermo Fischer). To ensure adequate oxygen supply for oxidative metabolism throughout the incubation period, flasks were filled with oxygen before being sealed. Glycolytic flux was measured by assaying the rate of ^3^H_2_O production from [3-^3^H]glucose by incubating cells with 5 μ Ci D-[3-^3^H]glucose in KRPG buffer per flask for 120 min, as previously described^32^. Incubations were then terminated with 0.2 ml 20% perchloric acid, and the cells were further incubated for 96 h with a microcentrifuge tube containing H_2_O, suspended above the cells to allow ^3^H_2_O equilibration. The ^3^H_2_O was then measured by liquid scintillation counting (Tri-Carb 4810 TR, PerkinElmer). Under these experimental conditions, 28% of the produced ^3^H_2_O was recovered and used for the calculations as previously established^7,32^.

### Lactate determination

Lactate released to the culture medium was determined as an estimation of glycolysis. To do so, the increments in absorbance of the culture medium samples were measured at 340 nm in a mixture containing 1 mM NAD^+^ and 22.5 units·ml^−1^ of lactate dehydrogenase in 0.25 M glycine/0.5 M hydrazine/1 mM EDTA at pH 9.5.

### Pyruvate dehydrogenase (PDH) activity

PDH was measured in 8 cm^2^ flasks of adherent cells at 60–70% confluence containing a central microcentrifuge tube with 0.8 ml benzethonium hydroxide (Sigma) for ^14^CO_2_ equilibration. Incubations were carried out in KRPG containing 5.5 mM D-glucose and 1 mM pyruvate at 37 °C in the air-thermostatized chamber of an orbital shaker (Forma Benchtop Orbital Shaker, Model 420, Thermo Fischer). To ensure adequate oxygen supply for oxidative metabolism throughout the incubation period, flasks were filled with oxygen before being sealed. To measure the carbon flux from pyruvate through the tricarboxylic acid cycle (TCA), cells were incubated in KRPG (5.5 mM D-glucose and 1 mM pyruvate), buffer supplemented with 0.5 μCi D-[1-^14^C]pyruvate for 90 min, as previously described^45^. Incubations were then terminated by the addition of 0.2 ml 20% perchloric acid (Merck Millipore) for 30 min before the benzethonium hydroxide (containing ^14^CO_2_) was removed, and the radioactivity was measured with a liquid scintillation analyzer (Tri-Carb 4810 TR, PerkinElmer). PDH activity was determined as the rate of [1-^14^C]pyruvate decarboxylation to ^14^CO_2_ through the TCA cycle.

### Mitochondrial membrane potential (Δψ_m_)

∆ψ_m_ was assessed using MitoProbe DiIC_1_(5) Assay Kit for flow cytometry (Molecular Probes Europe BV, Leiden, Netherlands) on a FACScalibur flow cytometer (15 mW argon ion laser tuned at 488 nm; CellQuest software, Becton Dickinson Biosciences). Δψ_m_ values were expressed as percentages, using carbonyl cyanide 4-(trifluoromethoxy)phenylhydrazone (CCCP; 10 μM) for 15 min to define the 0% Δψ_m_ values^21^ (see, also, Supplementary Fig. S4 for the flow cytometry workflow for mitochondrial membrane potential).

### Transient middle cerebral artery occlusion (MCAO)

Surgical endovascular insertion of a silicon-coated monofilament (602012PK10; Doccol Corporation, Sharon, MA, USA) was performed to induce transient middle cerebral artery occlusion (MCAO) for 30 minutes of ischemia, followed by filament removal to allow reperfusion^31,33^. Briefly, 10-weeks-old C57BL/6J mice were anesthetized with sevoflurane (4% for induction, 3% for maintenance) in a mixture of O_2_/N_2_O (30/70%). After surgical exposure of the right carotid artery tree, the filament was inserted through the external carotid artery and advanced through the internal carotid artery until it reached the middle cerebral artery. The regional cerebral blood flow was monitored during surgery with a laser Doppler probe (Moor Instruments, Devon, UK). After 30 minutes of ischemia, the filament was removed to allow reperfusion. AZ67 (60 mg/kg of body weight) or vehicle were administered in a bolus (200 µl) via the jugular vein immediately after reperfusion. Body temperature was maintained at 37 ± 0.5 °C using a heating pad connected to a rectal probe (BAT-12 thermometer; Physitemp Instruments Inc., Clifton, NJ, USA). Mice were then sutured and returned to the cages. Sham-operated mice underwent the same surgical procedure without middle cerebral artery occlusion.

### Rotarod analysis

An accelerating rotarod test was used to determine motor coordination. Animals were trained during the immediate three previous days of the MCAO surgery. The first day, mice stayed on the rotating rod at a constant speed of 4 rpm, and the remaining 2^nd^ and 3^rd^ day they stayed at an accelerating speed (4 to 40 rpm in 5 mins). For the test, which was performed 24 hours after the MCAO surgery, mice were subjected to three consecutive trials at the accelerating speed for 5 mins (at 15 mins intervals). The latency to fall was determined and expressed in seconds.

### Neurological severity score (NSS)

For the NSS test, mice were examined to assess the neurological status using a 0–5 grading scale as described^34^. Mice treated with the vehicle (DMSO) were scored 0, while dead mice were scored 5. The rest of the animals were examined and assigned a score for each of the following five items, following the test description^34^, namely (i) spontaneous activity, (ii) spontaneous rightward rotation, (iii) rightward rotation after grabbing the animal by the tail with both forelimbs placed at a platform, (iv) left forepaw extension deficit after grabbing the animal by the tail and (v) moving it closer to the platform.

### Infarct volume

Immediately after the rota-rod test, mice were euthanized by cervical dislocation after CO_2_ overdose, and the brain extracted and sliced in 2-mm coronal sections with a brain matrix on ice, which were used to determine the infarct volume after incubation of the slices in 2% (wt/vol) 2,3,5-triphenyltetrazolium chloride in phosphate-buffered saline (136 mM NaCl, 27 mM KCl, 7.8 mM Na_2_HPO_4_, 1.7 mM KH_2_PO_4_, pH 7.4) for 20 minutes at room temperature. Pictures of the brain sections were taken, and the images processed using the NIH image-processing package ImageJ 1.43n. Infarct volumes were determined by multiplying the selected infarcted area by the width of the slices. In order to correct the infarct volume by the edema, the ratio lesion volume of the ipsilateral (affected) *versus* that of the contralateral (unaffected) hemispheres was calculated. The percentage of infarct volume was calculated using the following formula: (infarcted volume corrected by edema × 100)/Infarcted hemisphere volume.

### Statistical analysis

Results from cultured cells were obtained from 3 independent culture preparations using 4–6 technical replicates per sample. Data were expressed as mean ± standard error of the mean (SEM) values, using as “n” the number of independent culture preparations. Statistical analysis of the results was performed by one-way or two-way analysis of variance (ANOVA), followed by the least significant difference multiple range test. In all cases, p < 0.05 was considered significant. Statistics were performed using Microsoft Excel or the IBM SPSS Statistics software.

## Supplementary information

Supplementary Information

Statistic Table 1

Statistic Table 2

Supplementary Data 1

Supplementary Data 2

## Data Availability

All data generated or analysed during this study are included in this published article.
