# Supplementary material for: Targeting PFKFB3 alleviates cerebral ischemia-reperfusion injury in mice
Source: Sci Rep. 2019 Aug 12;9:11670. doi: 10.1038/s41598-019-48196-z (PMC6691133; doi:10.1038/s41598-019-48196-z)
Supplement: Supplementary file 1 — Supplementary Information [file 41598_2019_48196_MOESM1_ESM.pdf]

SUPPLEMENTARY INFORMATION

Targeting PFKFB3 alleviates cerebral ischemia-reperfusion injury in mice

Olga Burmistrova[1*], Ana Olias-Arjona[2*], Rebeca Lapresa[2,3], Daniel Jimenez-Blasco[2,3,4], Tatiana Eremeeva[1], Dmitry Shishov[1], Sergei Romanov[5], Kristina Zakurdaeva[1], Angeles Almeida[2,3], Peter O. Fedichev[1#], Juan P. Bolaños[2,3,4#]

[1]Gero Discovery LLC, Moscow, Russia

[2]Institute of Functional Biology and Genomics (IBFG), Universidad de Salamanca, CSIC, Salamanca, Spain

[3]Institute of Biomedical Research of Salamanca (IBSAL), Hospital Universitario de Salamanca, Universidad de Salamanca, Spain

[4]Centro de Investigación Biomédica en Red de Fragilidad y Envejecimiento Saludable (CIBERFES), Madrid, Spain

[5]Nanosyn, Inc., Santa Clara, California, USA

[*]Shared first authorship

[#]Shared last and corresponding authorship (jbolanos@usal.es; peter.fedichev.gero.com)

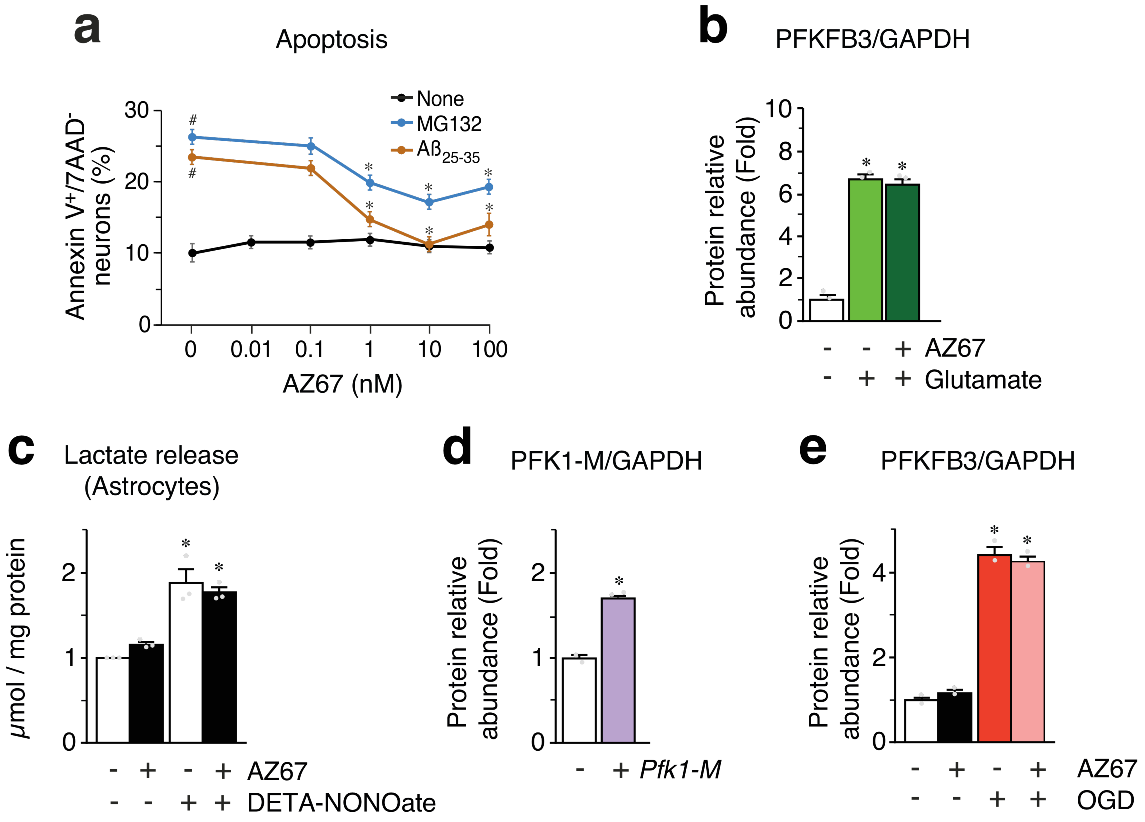

Supplementary Figure S1 (related to Figures 2 and 3)

(a) Incubation of mouse cortical primary neurons with AZ67 for 24 h revealed lack of toxicity. MG132 (10 µM; 24 h) or $Aß_{25-35}$ (10 µM; 24 h) increased neuronal apoptosis (compare MG132 or $Aß_{25-35}$ *versus* none values at 0 nM AZ67). Incubation of neurons with AZ67 together with MG132 or $Aß_{25-35}$, for 24 h, dose-dependently prevented apoptosis. (Related to Figure 2).

(b) Relative quantification of the western blots represented in Figure 2a.

(c) Incubation of astrocytes with AZ67 for 24 h revealed no effect on lactate release. Treatment of astrocytes with the nitric oxide donor, DETA-NONOate (0.5 mM) increased lactate released after 24 h of incubation. However, incubation of astrocytes with AZ67 was unable to prevent the DETA-NONOate-mediated increase in lactate released.

(d) Relative quantification of the western blots represented in Figure 2g**.**

(e) Relative quantification of the western blots represented in Figure 3a.

See also Supplementary Data 2 and Statistics Table 2.

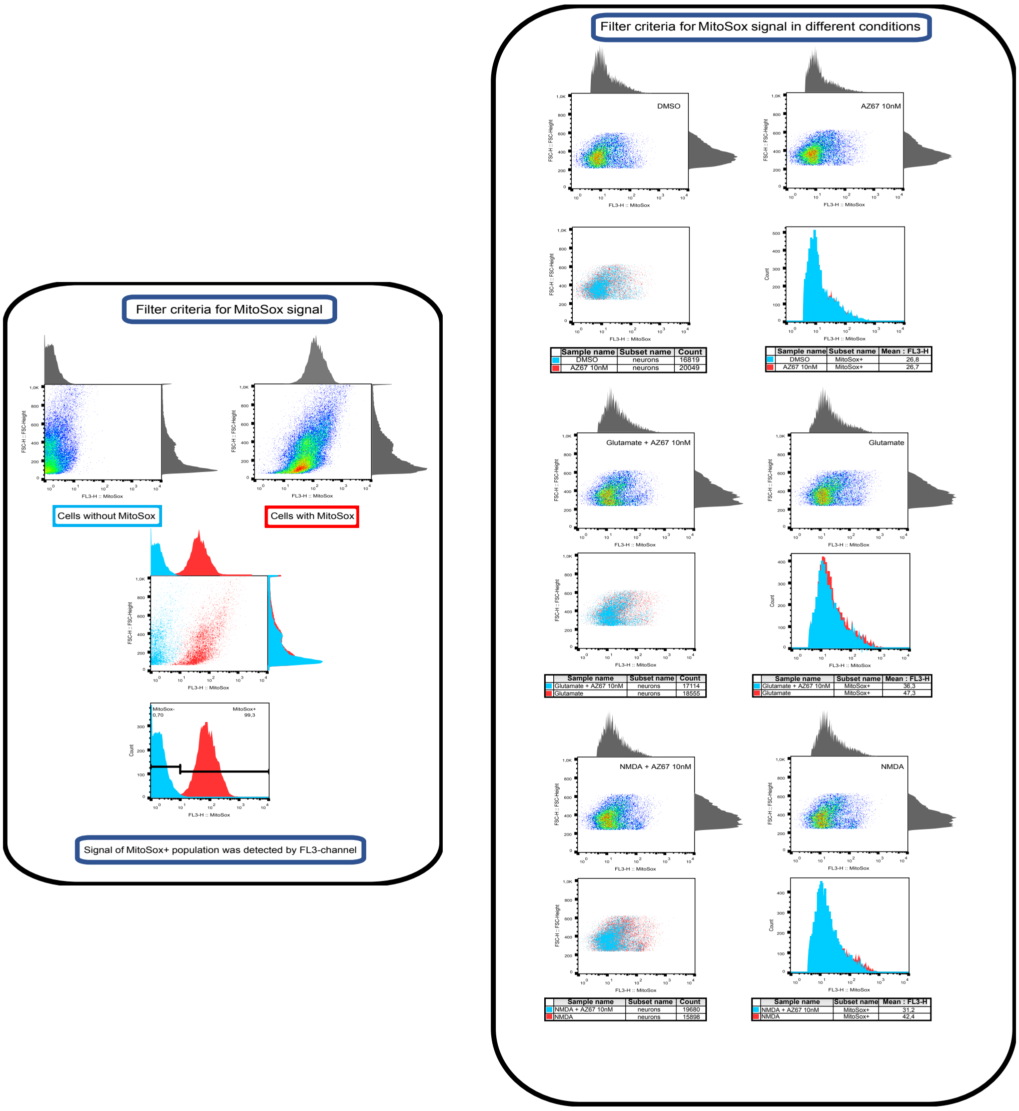

Supplementary Figure S2 (related to Figures 2 and 3).

Extended data for flow cytometry workflow for MitoSox.

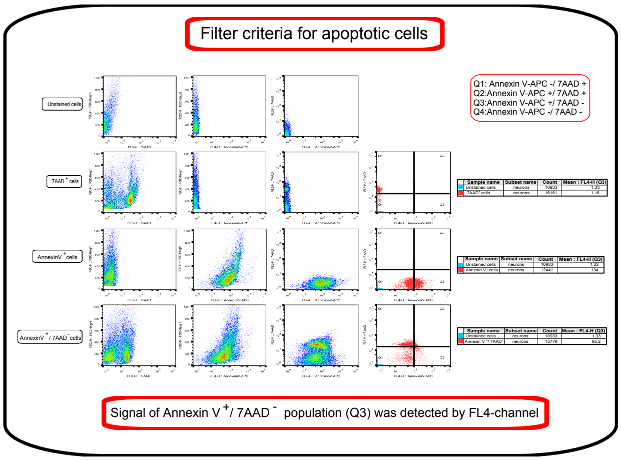
Filter criteria for apoptotic cells
Q1: Annexin V-APC -/ 7AAD +
Q2:Annexin V-APC +/ 7AAD +
Q3:Annexin V-APC +/ 7AAD -
Q4:Annexin V-APC -/ 7AAD -
Unstained cells
7AAD + cells
AnnexinV + cells
AnnexinV + / 7AAD - cells
Signal of Annexin V +/ 7AAD - population (Q3) was detected by FL4-channel

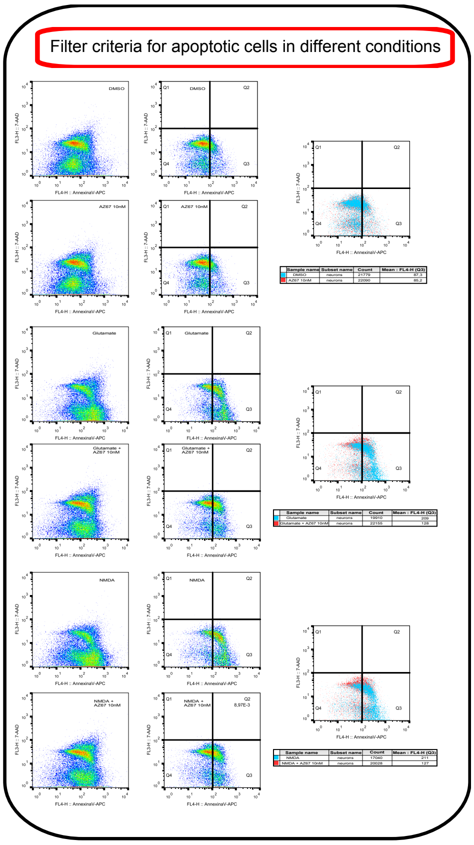
Filter criteria for apoptotic cells in different conditions
DMSO
AZ67 10nM
Glutamate
Glutamate + AZ67 10nM
NMDA
NMDA + AZ67 10nM
FL3-H :: 7AAD
FL4-H :: AnnexinV-APC

Supplementary Figure S3 (related to Figures 2 and 3).

Extended data for flow cytometry workflow for apoptosis.

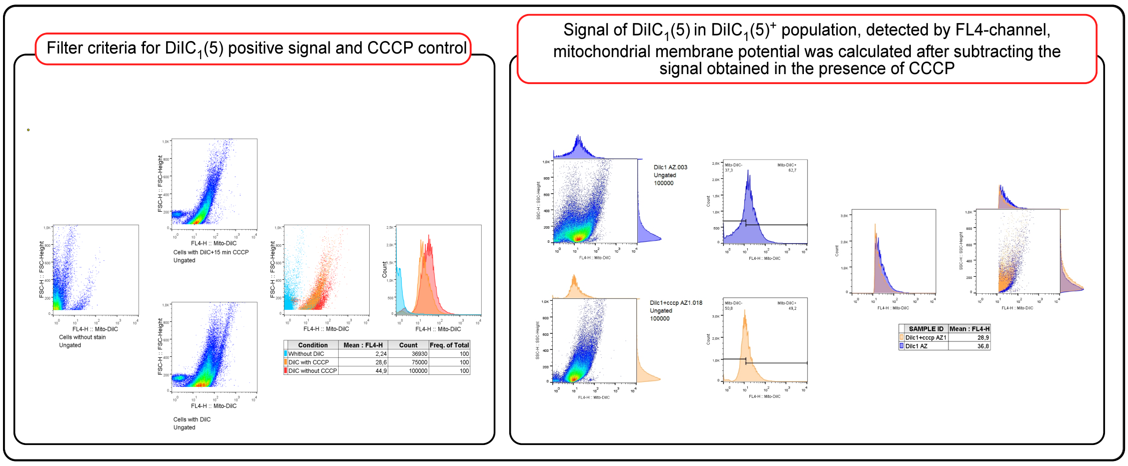

Supplementary Figure S4 (related to Figure 3).

Extended data for flow cytometry workflow for mitochondrial membrane potential.

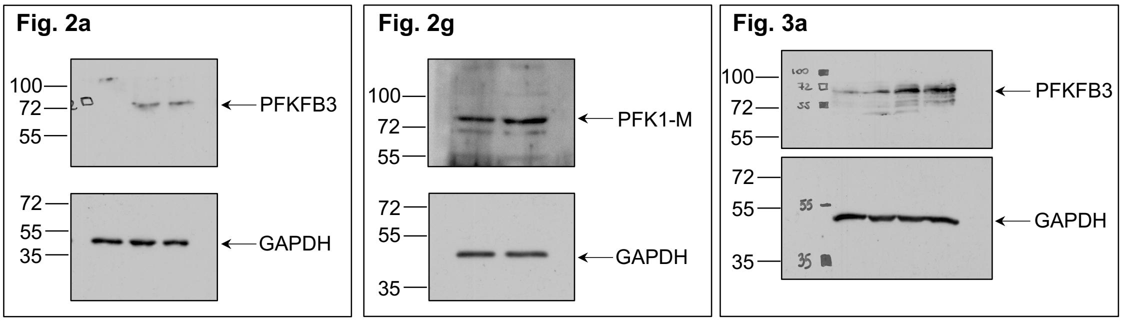

Supplementary Figure S5 (related to Figures 2 and 3).

Original western blot replicas.
